# Structural basis of tethered agonism and G protein coupling of protease-activated receptors

**DOI:** 10.1038/s41422-024-00997-2

**Published:** 2024-07-12

**Authors:** Jia Guo, Yun-Li Zhou, Yixin Yang, Shimeng Guo, Erli You, Xin Xie, Yi Jiang, Chunyou Mao, H. Eric Xu, Yan Zhang

**Affiliations:** 1 Department of Pharmacology and Department of Pathology of Sir Run Run Shaw Hospital, Zhejiang University School of Medicine, Hangzhou, Zhejiang China; 2 Liangzhu Laboratory, Zhejiang University, Hangzhou, Zhejiang China; 3 Department of General Surgery, Sir Run Run Shaw Hospital, Zhejiang University School of Medicine, Hangzhou, Zhejiang China; 4 Center for Structural Pharmacology and Therapeutics Development, Sir Run Run Shaw Hospital, Zhejiang University School of Medicine, Hangzhou, Zhejiang China; 5 CAS Key Laboratory of Receptor Research, Center for Structure and Function of Drug Targets, Shanghai Institute of Materia Medica, Chinese Academy of Sciences, Shanghai, China; 6 University of Chinese Academy of Sciences, Beijing, China; 7 Lingang Laboratory, Shanghai, China; 8 Zhejiang Research and Development Engineering Laboratory of Minimally Invasive Technology and Equipment, Zhejiang University, Hangzhou, Zhejiang China; 9 MOE Frontier Science Center for Brain Research and Brain-Machine Integration, Zhejiang University School of Medicine, Hangzhou, Zhejiang China

**Keywords:** Cryoelectron microscopy, Extracellular signalling molecules

## Abstract

Protease-activated receptors (PARs) are a unique group within the G protein-coupled receptor superfamily, orchestrating cellular responses to extracellular proteases via enzymatic cleavage, which triggers intracellular signaling pathways. Protease-activated receptor 1 (PAR1) is a key member of this family and is recognized as a critical pharmacological target for managing thrombotic disorders. In this study, we present cryo-electron microscopy structures of PAR1 in its activated state, induced by its natural tethered agonist (TA), in complex with two distinct downstream proteins, the G_q_ and G_i_ heterotrimers, respectively. The TA peptide is positioned within a surface pocket, prompting PAR1 activation through notable conformational shifts. Contrary to the typical receptor activation that involves the outward movement of transmembrane helix 6 (TM6), PAR1 activation is characterized by the simultaneous downward shift of TM6 and TM7, coupled with the rotation of a group of aromatic residues. This results in the displacement of an intracellular anion, creating space for downstream G protein binding. Our findings delineate the TA recognition pattern and highlight a distinct role of the second extracellular loop in forming β-sheets with TA within the PAR family, a feature not observed in other TA-activated receptors. Moreover, the nuanced differences in the interactions between intracellular loops 2/3 and the Gα subunit of different G proteins are crucial for determining the specificity of G protein coupling. These insights contribute to our understanding of the ligand binding and activation mechanisms of PARs, illuminating the basis for PAR1’s versatility in G protein coupling.

## Introduction

**Fig. 1:**
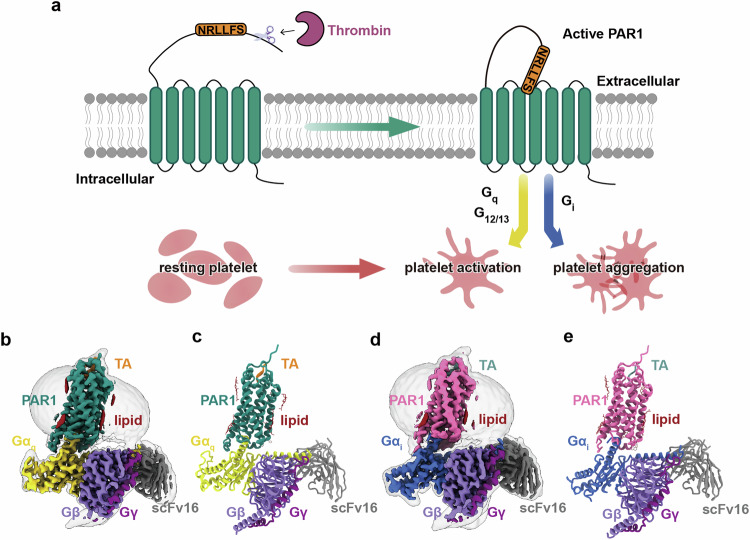
Activation of PAR1 and cryo-EM structures of TA–PAR1–G_q_ and TA–PAR1–G_i_ complexes. **a** PAR1 is cleaved by thrombin at the N-terminus which exposes the TA, SFLLRN, which can bind and activate PAR1, arousing several G protein signaling pathways, including G_q_, G_12/13_ and G_i_ leading to platelet activation and aggregation. **b**, **c** Cryo-EM map (**b**) and model (**c**) of the TA–PAR1–G_q_ complex. The TA is shown in orange, PAR1 in teal, Gα_q_ in yellow, Gβ in purple, Gγ in dark magenta, and scFv16 in gray. **d**, **e** Cryo-EM map (**d**) and model (**e**) of the TA–PAR1–G_i_ complex. The TA is shown in light green, PAR1 in pink, Gα_i_ in blue, Gβ in purple, Gγ in dark magenta, and scFv16 in gray.

Protease-activated receptors (PARs) are a unique subset of G protein-coupled receptors (GPCRs) activated by proteolytic cleavage.^1–6^ PAR1, primarily located on platelets, endothelial cells, and smooth muscle cells,^7^ plays a pivotal role in regulating hemostasis and thrombosis.^8,9^ The increased generation of thrombin and expression of PAR1 within atherosclerotic plaques, thrombi, and post-vascular injury highlights PAR1 as a promising therapeutic target for hypercoagulable states. Initially identified as a thrombin receptor, PAR1 is transiently activated by thrombin, which cleaves the receptor, revealing a new N-terminus. The first six amino acids of this N-terminus (SFLLRN) act as a tethered agonist (TA)^2^ (Fig. 1a). Synthetic peptides mimicking this TA can also activate PAR1.^10^ Mutagenesis studies indicate that the TA occupies a superficial binding pocket^10,11^ to activate PAR1, yet the details of this interaction and activation process remain elusive. PAR1 activation through TA can signal through a spectrum of G proteins, including G_q/11_, G_12/13_ and G_i_.^3,12–14^ Activation through G_q/11_ and G_12/13_ initiates platelet activation,^15,16^ and the engagement of G_i_ provokes a decrease in intracellular cyclic AMP levels, thereby fostering platelet aggregation^17^ (Fig. 1a). The FDA-approved small-molecule antagonist, vorapaxar, effectively and irreversibly inhibits various downstream signaling pathways of PAR1; however, its usage is limited due to the increased risk of bleeding and intracranial hemorrhage.^18–21^ To avoid the disadvantage of the orthosteric antagonist, an increasing number of allosteric modulators, including parmodulins and pepducins, are designed to target the intracellular domain of PAR1,^22–25^ with the aim of disrupting or inhibiting G protein binding. Ligands that possess the ability to selectively inhibit particular signaling pathways are of particular necessity, yet the unknown mechanisms of G protein coupling selectivity of PAR1 have posed significant challenges for drug development. Currently, only inactive structures of PAR1 and PAR2 have been reported.^10,26^ The lack of active structures hampers the understanding of how PARs recognize the TA, initiate activation, and bind G proteins. In this study, we present two cryo-electron microscopy (cryo-EM) structures of TA-activated PAR1 in complex with heterotrimeric G_q_ or G_i_ protein (Fig. 1b–e). The elucidated structures provide insights into the distinctive ligand recognition mode, the mechanism of receptor activation, and the basis for the G protein promiscuity and selectivity of PAR1.

## Results

### Cryo-EM studies of PAR1 signaling complexes

We used single-particle cryo-EM to determine the structure of PAR1 activated by the TA in complex with G_q_ or G_i_ protein, respectively. The N-terminal 41 amino acids of PAR1 were removed to unmask the TA, and the C-terminal region after helix 8 was deleted to improve receptor stability.^10^ Hemagglutinin (HA) signal peptide was fused into the N-terminus of PAR1, and a double maltose-binding protein (MBP) tag was introduced to the C-terminus of PAR1 to facilitate purification (Supplementary information, Fig. S1a). An engineered Gα_q_ chimera^27^ was used. Gα_i_ contains two dominant-negative mutations (G203A and A326S).^28^ A NanoBiT tethering strategy^29^ was adopted to increase the stability of the complexes (Supplementary information, Fig. S1a–c). Human PAR1, engineered Gα, rat Gβ, bovine Gγ, and scFv16 were co-expressed in Sf9 insect cells.

The structure of the TA–PAR1–G_q_ complex was determined with 723,778 final particles from 3,944,919 initial particles to a global resolution of 3.0 Å. The structure of the TA–PAR1–G_i_ complex was determined with 162,724 particles from 5,979,500 particles to a global resolution of 3.2 Å (Supplementary information, Fig. S1d–i and Table S1). We can unambiguously model the TA, most residues of PAR1 from amino acid 82–397, and G_q_ or G_i_ heterotrimer except the α-helical domain in Gα components (Supplementary information, Fig. S2). The reported positive allosteric modulator of PAR1, GB83,^30,31^ was added during the purification of both G_q_ and G_i_ complexes, but was not visible in the final electron density map.

**Fig. 2:**
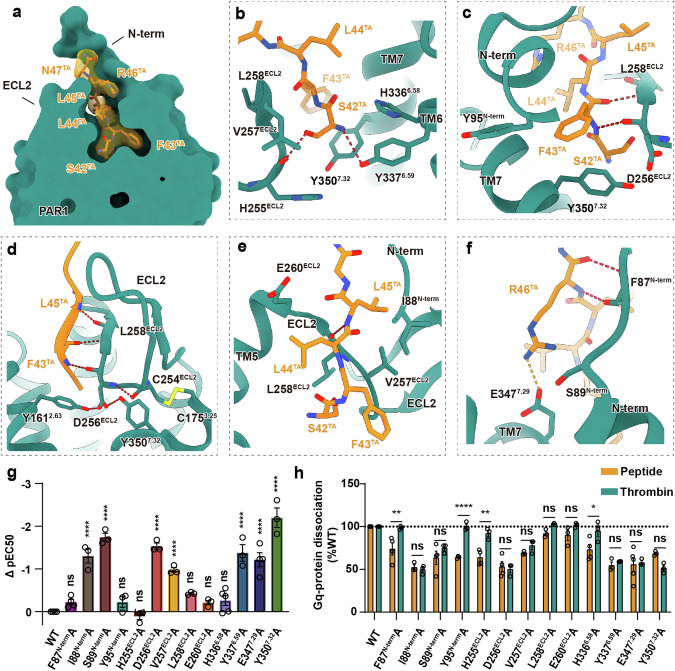
Intramolecular interactions of the TA. **a** The TA-binding pocket in PAR1. **b**–**f** Detailed interactions between the TA and the transmembrane core of PAR1. Side chains of residues are displayed in sticks. Hydrogen bonds are depicted as red dashed lines, the salt bridge is depicted as a orange dashed line. **g** Effects of mutations of residues in the binding pocket on Gα_q_–Gγ dissociation induced by the synthesized TA peptide. ΔpEC_50_ represents the difference between pEC_50_ values of the mutant PAR1 receptor and the wild-type (WT) receptor. Data are presented as means ± SEM of more than three independent experiments performed in technical triplicate. NS, *P* ≥ 0.05, *P < 0.05, **P < 0.01, ***P < 0.001 and ****P < 0.0001 by one-way ANOVA followed by Fisher’s LSD multiple comparisons test compared with WT PAR1. **h** Comparison between the efficacy of PAR1 signaling activated by thrombin and the synthesized TA peptide. The efficacy is defined as the range between the maximal response (*E*_max_) and the vehicle baseline (no agonist). Data are presented as means ± SEM of more than three independent experiments performed in technical triplicate. NS, *P* ≥ 0.05, *P < 0.05, **P < 0.01, ***P < 0.001 and ****P < 0.0001 by two-way ANOVA. A detailed statistical evaluation is provided in Supplementary information, Fig. S4 and Tables S3, S4.

The receptor assumes a typical seven transmembrane architecture in both structures, with all extracellular loop (ECL) and intracellular loop (ICL) densities being clearly distinguishable, except for ICL3 of the receptor in the TA–PAR1–G_i_ complex. PAR1 in both G_i_ and G_q_ complexes adopts a quite similar pose, with the all-atom root-mean-square deviation (RMSD) being 0.834 Å for the receptor. The structure of the TA–PAR1–G_q_ complex with a higher resolution is used for further detailed analysis of the TA-binding pocket and activation mechanism of PAR1. The linker from P48^N-term^ to L84^N-term^ between the TA and the N-terminus of the receptor lacks density, possibly due to its highly flexible nature. The binding of TA to the extracellular segment of the receptor results in an extended helix at the N-terminus of the receptor (Fig. 1b–e). TA is observed to insert into the receptor at a 75-degree angle relative to the membrane, and forms a parallel β-sheet with ECL2 (Fig. 2a). While the binding of TA does not induce significant rotation and outward movement of TM6, a pocket satisfying G protein binding is still present in the intracellular region.

### TA binding of PAR1

Our maps revealed well-resolved densities for the native TA peptides bound within the orthosteric binding site composed of the N-terminal region, ECL2, and the seven transmembrane bundle (Fig. 2). Compared with the inactive-state structure,^10^ the active-state structure reveals a stretched-out density at the N-terminus (Fig. 2a), with the N-terminus forming hydrogen bonds with the TA (Fig. 2f). Similar to other peptide receptors, ECL2 forms a β hairpin structure^32^; L44^TA-03^ and L45^TA-04^ (the superscript numbers indicate positions within the TA) in the middle part of the TA form an additional β-sheet with L258^ECL2^ in ECL2 of the receptor (Fig. 2d; Supplementary information, Fig. S3a). The N-terminus of TA inserts into the receptor, with the S42^TA-01^ making the deepest penetration, and the following Cα chain adopting a snake-like conformation as it enters the ligand-binding pocket (Fig. 2a). The TA has a superficial binding pocket with a distance of 18.4 Å to the 6.48 position of the toggle switch residue in TM6 (Supplementary information, Fig. S3b), which is different from most class A GPCRs where agonist binding directly turns the side chain of the established toggle switch amino acid W^6.48^. In our model, five amino acids of TA establish stable interactions with the receptor, encompassing hydrogen bonds, hydrophobic and van der Waals interactions, and a salt bridge. Specifically, S42^TA-01^ forms hydrogen bonds with the main chain of H255^ECL2^ and the side chain of Y337^6.59^, as well as van der Waals interactions with the side chains of V257^ECL2^, L258^ECL2^, and Y350^7.32^ (Fig. 2b; Supplementary information, Fig. S3a and Table S2). F43^TA-02^ forms hydrogen bonds with the main chains of D256^ECL2^ and L258^ECL2^ (Fig. 2c; Supplementary information, Fig. S3a and Table S2), while L44^TA-03^ and L45^TA-04^ contribute to a β-sheet secondary structure with V257^ECL2^ and L258^ECL2^ (Fig. 2d, e; Supplementary information, Fig. S3a and Table S2). The formation of a disulfide bond between C254^ECL2^ and C175^3.25^ restricts the movement of the β-hairpin formed by T243^ECL2^ to C254^ECL2^ (Fig. 2d). Additionally, the hydrogen bond between the main chains of C254^ECL2^ and D256^ECL2^, and those between the side chain of D256^ECL2^ and the side chains of Y161^2.63^ and Y350^7.32^ contribute to the stability of ECL2 (Fig. 2d), ensuring the formation of the interactions between the ECL2 and the TA. Therefore, the Ala mutation of D256^ECL2^ significantly diminishes the potency of TA binding by 33-fold (Fig. 2g; Supplementary information, Fig. S4 and Table S3). Additionally, Y350^7.32^ forms van der Waals interactions with S42^TA-01^ and engages in π–π stacking with F43^TA-02^, contributing to the stability of the ECL2 position; its mutation to Ala resulted in a substantial 158-fold decrease in the potency of the TA at PAR1 (Fig. 2g; Supplementary information, Fig. S4 and Table S3). The conservation of the disulfide bond formed by C254^ECL2^ and C175^3.25^ in peptide receptors, as well as the absolute conservation of D256^ECL2^, Y161^2.63^, and Y350^7.32^ across the PAR family (Supplementary information, Fig. S3c), suggests a common strategy for restricting the position of ECL2 in this receptor subfamily. R46^TA-05^ forms hydrogen bonds with the N-terminus and a salt bridge with E347^7.29^, along with polar interactions with S89^N-term^ (Fig. 2f; Supplementary information, Fig. S3a and Table S2). Mutagenesis studies of S89^N-term^, V257^ECL2^, Y337^6.59^, and E347^7.29^ have also demonstrated significant effects on agonist potency (Fig. 2g; Supplementary information, Fig. S4 and Table S3). We also investigated the functional impacts of mutations in the binding pocket on the PAR1 basal activity elicited by its tethered N-terminus, which revealed a similar pattern to that observed using synthetic peptides (Fig. 2h; Supplementary information, Table S4). However, we did observe slight differences for the F87^N-term^A, Y95^N-term^A, H255^ECL2^A, and H336^6.58^A mutants (Fig. 2h; Supplementary information, Table S4). In the functional assay using synthetic TA peptides, these four mutants exhibited no significant change in potency though decreased efficacy, whereas in the N-terminus activation experiment, they showed no significant change in efficacy (Fig. 2g, h; Supplementary information, Tables S3, S4). Previous NMR structural study of PAR1 S42^TA-01^–S103^N-term^ segment revealed interactions between the TA and the N-terminal residues P85^N-term^–S89^N-term^.^33^ The presence of this additional TA-binding site suggests that the binding of TA to the receptor likely initiates at an upper position on the N-terminus of the receptor before proceeding into the final deeper binding pocket. Furthermore, the structures of TA-activated PAR2, PAR3, and PAR4 were predicted using our determined active PAR1 as a homologue template. The predicted structures demonstrate that L^TA-02^–I^TA-03^–G^TA-04^ in PAR2, G^TA-04^–A^TA-05^ in PAR3 and P^TA-02^–G^TA-03^ in PAR4 (Supplementary information, Fig. S3c) all exhibit potential for forming a β-sheet, indicating a potentially shared mechanism for TA–ECL2 interactions within the PAR family and revealing a common TA binding mode of the PAR receptors.

The adhesion GPCRs (aGPCRs) are also known to be activated by a TA called the Stachel sequence, which becomes exposed through either autoproteolysis or mechanical force. The TA of aGPCRs typically comprises 15 conserved amino acids,^34–39^ exceeding the length of the TA for the PAR family. The amino acid at the C-terminal end of the Stachel sequence is directly associated with the extracellular N-terminal end of TM1 in aGPCRs,^34–39^ whereas a substantial linker of 50 amino acids exists between the TA and TM1 in PAR1. The intramolecular interactions formed between the Stachel sequence of aGPCRs and their receptors involve a greater extent of the transmembrane domain (TMD), particularly TM2, TM3, TM5, TM6, and TM7,^34–39^ resulting in a 16-Å deeper binding position compared to PAR1 (Supplementary information, Fig. S5a). When the Stachel sequence binds to aGPCR, it adopts an α-helical conformation (Supplementary information, Fig. S5a), whereas the TA of PAR1 forms a sheet. Hence, aGPCRs possess a wide, U-shaped binding pocket,^34–39^ whereas PAR1 features an elongated pocket (Supplementary information, Fig. S5a). The TA of PAR1 is predominantly positively charged, establishing strong electrostatic attraction with negatively charged regions such as ECL2 on the receptor (Supplementary information, Fig. S5b). In contrast, the lower rim of the Stachel sequence in aGPCRs primarily relies on hydrophobic interactions to firmly bind the receptor^34–39^ (Supplementary information, Fig. S5b, c). These distinct features highlight a novel, superficial β-sheet conformation of the TA in PARs, which contrasts with the deeply embedded α-helical conformation of the TA observed in aGPCRs.

### Activation mechanism of PAR1

**Fig. 3:**
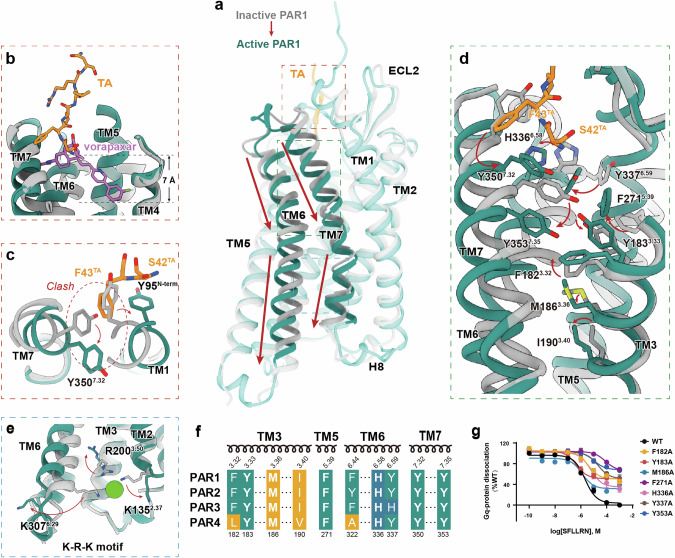
Activation mechanism of PAR1. **a** Superposition of the TA-bound PAR1 structure (teal) with the inactive PAR1 structure (gray) (PDB: 3VW7^10^). **b** Comparison of the binding pocket of the TA and the antagonist vorapaxar in PAR1. **c** Conformational changes of Y95N^N-term^ and Y350^7.32^ induced by F43^TA-02^ upon TA binding. **d** The rearrangement of local residues in the backbone of TMD in TA-bound PAR1 compared to the inactive structure shows the cascade changes of residues H336^6.58^, Y337^6.59^, Y353^7.35^, F271^5.39^, Y183^3.33^, F182^3.32^, M186^3.36^, and I190^3.40^. **e** The “K-R-K” motif involved in activation signal transmission of PAR1. The “K-R-K” motif consists of K135^2.37^, R200^3.50^, and K307^6.29^ of PAR1. **f** Sequence alignment of residues involved in the activation of PAR family receptors. **g** Mutations of F182^3.32^, Y183^3.33^, M186^3.36^, F271^5.39^, H336^6.58^, Y337^6.59^, and Y353^7.35^ in PAR1 decreased the activation potency induced by the TA peptide. Data represent the means ± SEM from three biologically independent experiments performed in triplicate. A detailed statistical evaluation is provided in Supplementary information, Table S5.

Upon superimposing the PAR1 structures in the active and inactive states, the TA–PAR1–G protein complex exhibits an elongated N-terminus and clear densities of the ICLs (Fig. 3a; Supplementary information, Fig. S2). Noteworthy conformational alterations are particularly evident in ECL2, the extracellular end of TM5, TM6 and TM7, as well as the intracellular end of TM1, TM6 and TM7 (Supplementary information, Fig. S6a, b). The activation of class A GPCRs typically results in the expansion of the cytoplasmic pocket through the displacement and rotation of TM6 on the intracellular side of the receptor to facilitate G protein binding.^40–42^ Nevertheless, this characteristic is not observed in the comparison between the active and inactive structures of PAR1. Instead, a downward slide of the TM6 and TM7 helices is established (Fig. 3a). The intracellular end of TM5 may also undergo alterations during the process of activation,^43^ though only minor conformational changes are revealed by structural comparison (Fig. 3a; Supplementary information, Fig. S6a, b). The comparison of the binding pockets of the TA and the antagonist vorapaxar reveals that the TA occupies a pocket shallower by 7 Å (Fig. 3b). Nevertheless, despite these disparities, the TA can induce effective propagation of the activation signal by eliciting a cascade of conformational changes in the receptor, particularly in TM6 and TM7 (Fig. 3a). Upon engagement with the TA, the downward movement of H336^6.58^ and Y337^6.59^ triggers a corresponding downward displacement of TM6 to accommodate the interaction with S42^TA-01^ (Fig. 3d). The insertion of F43^TA-02^ induces steric hindrance, leading to the repositioning of Y95^N-term^ away from the ligand-binding pocket, consequently inducing conformational changes in the upper region of TM1 (Fig. 3c, d). Additionally, F43^TA-02^ exerts a downward force on Y350^7.32^, causing a global shift in the position of TM7 (Fig. 3c, d). The displacement of TM7 and Y350^7.32^ induces a sequential shift of the positions of the bulky side chains ranging from Y353^7.35^, F271^5.39^, Y183^3.33^, F182^3.32^ to M186^3.36^, ultimately resulting in the shift of I190^3.40^ (Fig. 3d). This cascade of events transmits the activation signal to the connector region which is contiguous to the G protein-binding interface and consists of the P^5.50^I^3.40^F^6.44^ motif, D^7.49^P^7.50^xxY^7.53^ motif, and D^3.49^R^3.50^F^3.51^ motif (Supplementary information, Fig. S6c–e). Mutations of these residues substantially hindered the activation of PAR1 and disrupted G protein signaling (Fig. 3g; Supplementary information, Table S5).

Notably, in the structure of the inactive PAR1 (PDB: 3VW7^10^), there is an anion that is stabilized by the surrounding basic residues K135^2.37^, R200^3.50^ and K307^6.29^, potentially impeding the binding of the G protein. Upon receptor activation, the side chains of these basic residues undergo substantial conformational changes (Fig. 3e), disrupting the stabilization of the anion in the intracellular pocket and allowing the binding of the α5 helix of the G protein. Mutations of these residues significantly hampered G protein signaling^44^ (Supplementary information, Tables S6 and S7). The residues involved in the activation signal transduction are highly conserved within the PAR family (Fig. 3f; Supplementary information, Fig. S6f), suggesting that this unconventional activation mechanism may be embraced by other receptors in this family. It is noteworthy that the substitution of the residues at positions 3.32, 3.40 and 6.44 with less bulky residues in PAR4 (Fig. 3f) may hamper the effective activation of the receptor, possibly contributing to its slower response in platelets.^45^ Together, the interactions with the TA provoke the slide of TM6 and TM7 relative to other transmembrane helices and the propagation of the activation signal by the cascade of aromatic residues, demonstrating how the TA binding at a pocket with a considerable distance from the TMD induces significant changes in PAR1.

PAR1 is closely related to purinergic receptors.^46,47^ Specifically, P2Y purinoceptor 1 (P2Y_1_R) and P2Y_12_R play crucial roles in the platelet thrombosis,^46,47^ exhibiting highly functional similarities to PAR1. However, although P2Y_1_R and P2Y_12_R exhibit similar active structures to that of PAR1, P2Y receptors follow a canonical activation mechanism of class A GPCRs, characterized by a pronounced rotation of TM5 and an outward displacement of TM6 (Supplementary information, Fig. S6g–j). This activation mechanism distinctly differs from what is observed in PAR1, highlighting divergent molecular strategies between receptor families involved in thrombotic processes.

### ICL2 and ICL3 mediate G protein recognition and specificity in PAR1

**Fig. 4:**
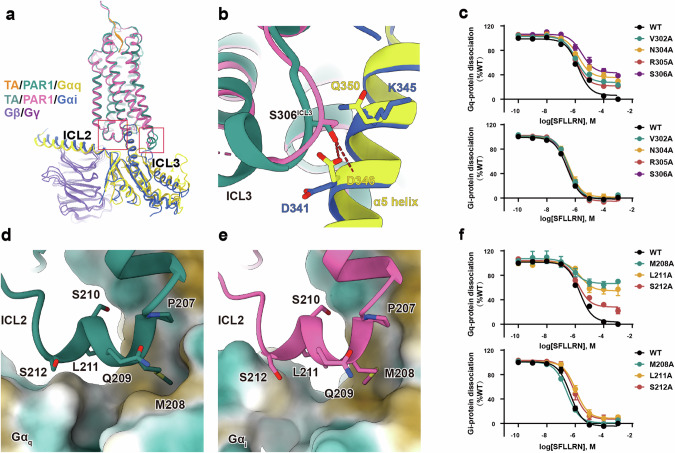
G protein selectivity mediated by ICLs of PAR1. **a** Structural superposition of the TA–PAR1–G_q_ and TA–PAR1–G_i_ complexes. **b** Differences in the interactions between ICL3 with Gα_q_ and Gα_i_. **c** Effects of mutations in ICL3 on Gα_q_–Gγ and Gα_i_–Gγ  dissociation induced by the synthesized TA peptide. **d**, **e** The positioning of PAR1 ICL2 within the hydrophobic cavities of Gα_q_ (**d**) and Gα_i_ (**e**), respectively. The light yellow color indicates hydrophobicity. **f** Effects of mutations in ICL2 on Gα_q_–Gγ and Gα_i_–Gγ dissociation induced by the synthesized TA peptide. A detailed statistical evaluation is provided in Supplementary information, Tables S6 and S7.

PAR1 can couple with various G proteins to elicit different signals, and the precise mechanism for regulation of downstream signaling pathways remains poorly understood, which hinders the development of novel molecules to achieve selective modulation. The overall structure of the PAR1–G_q_ complex is similar to that of the PAR1–G_i_ complex (Fig. 4a). PAR1 interacts with the G protein through TM2, TM3, TM6, TM7, helix 8, ICL2, and ICL3 (Fig. 4a). The active conformation and the G protein-coupling interface of PAR1 resemble those in the CCK_A_R–G protein complexes (Supplementary information, Fig. S7). Moreover, in the PAR1–G_q_ complex, the ICL1 of PAR1 and the β16 strand of Gα_q_ also participate in the formation of interactions (Supplementary information, Fig. S8b, c, e, f). The solvent-accessible surface area buried between PAR1 and Gα_q_ is 1145.6 Å^2^, larger than 1044.3 Å^2^ in the PAR1–G_i_ complex, implying more extensive interactions between G_q_ and the receptor. In the G_q_ complex structure, the ICL3 of the receptor adopts a secondary structure of α-helix (Supplementary information, Fig. S2a), which is also observed in the CCK_A_R–G_q_ complex.^48^ The residue S306^ICL3^ engages in polar interactions with Q350^H5.17^ in the α5 helix of Gα_q_ and forms two pairs of hydrogen bonds with the main chain and side chain of residue D346^H5.13^ in the α5 helix of Gα_q_ (Fig. 4b; Supplementary information, Fig. S8a). In contrast, the PAR1–G_i_ complex exhibits insufficient density in the middle part of ICL3 of the receptor to support model building, suggesting the absence of stable interactions with other residues in this region. The side chains of K345^H5.17^ and D341^H5.13^ in the α5 helix of Gα_i_ do not interact with S306^ICL3^ (Fig. 4b; Supplementary information, Fig. S8d). To validate the role of these residues in G protein binding, individual residues on ICL3 were mutated to alanine. The results of alanine screening demonstrate that the S306^ICL3^A mutation has the most pronounced impact on the efficacy of PAR1 coupling to G_q_, reducing it by 30%, and does not have a significant effect on the coupling of G_i_ (Fig. 4c; Supplementary information, Fig. S9 and Tables S6, S7), indicating a significant role of S306^ICL3^ in G_q_ coupling. Furthermore, mutations of other residues on ICL3 exhibit varying degrees of impact on the efficacy of G_q_ signaling but do not have a substantial effect on G_i_ signaling (Fig. 4c; Supplementary information, Fig. S9 and Tables S6, S7), suggesting that the formation of the ICL3 helix also influences G_q_ signaling. In addition, distinct effects on G protein signaling were also observed upon alanine substitution in ICL2 (Fig. 4f; Supplementary information, Fig. S9 and Tables S6, S7). Mutations of M208^ICL2^A, L211^ICL2^A, and S212^ICL2^A exert a vital impact on the efficacy of G_q_ signaling, while demonstrating minimal effects on G_i_ binding. The M208^ICL2^A variant demonstrates the most pronounced difference, exhibiting a 58% reduction in G_q_ signaling efficacy but no difference in G_i_ signaling (Fig. 4f; Supplementary information, Fig. S9 and Tables S6, S7). Comparison of the two structures revealed that, despite the overall similar conformation of ICL2 (Supplementary information, Fig. S2), the PAR1–G_q_ structure exhibits a closer and larger hydrophobic interaction interface between ICL2 and the G protein (Fig. 4d, e). The residue at position 34.51 in ICL2, which corresponds to M208^ICL2^, has been extensively documented to be critical for G_q_ or G_s_ coupling^49–52^ but non-essential for G_i/o_ coupling.^53–55^ Furthermore, the ICL3 plays a pivotal role in the association of certain receptors (e.g., PAR1 and CCK_A_R) with G_q_,^48^ while it contributes more significantly to G_i_ coupling in some other receptors, such as SSTR2,^56,57^ demonstrating the variable roles of ICL3 in G protein coupling. The observation that conformational changes in ICL3 have a more pronounced effect on G_q_ signaling than on G_i_ signaling may explain why parmodulins, potentially interacting with the ICL3 of PAR1, can selectively inhibit PAR1-mediated G_q_ signaling.^24,58,59^ Furthermore, the distinct roles of ICL2 in G_q_ and G_i_ coupling suggest that ICL2 could serve as a novel binding site for the development of modulators that specifically target individual G protein signaling pathways.

## Discussion

The PAR family represents a unique class of GPCRs distinguished by a protease-mediated revelation of endogenous agonists. The superficial TA-binding pocket of this receptor family is characterized by ECL2 that facilitates ligand binding through a β-sheet-like interaction with the TA, stabilized by a disulfide bond between C254^ECL2^ and C175^3.25^, in conjunction with hydrogen bonds between C254^ECL2^, D256^ECL2^, Y161^2.63^ and Y350^7.32^. This configuration underscores a novel mode of TA binding distinct from that in aGPCRs.

The activation of PAR1 is not by direct alterations to the toggle switch, but through the slide of TM6 and TM7 together with the rotation of a conserved cluster of aromatic residues, representing a defining characteristic of the PAR family. These investigations have unraveled the molecular basis underlying the unique mechanism of agonist interaction and the associated conformational changes upon receptor activation within this family, providing a molecular framework for the comprehension of the physiological functions of PARs.

Structural elucidation of the two G protein-bound active states of PAR1 along with functional assays has yielded a more detailed and expansive view of the importance of the ICLs in selective G protein coupling, thereby not only facilitating the dissection of the molecular mechanisms dictating downstream signaling specificity but also supporting the rational design of precise allosteric modulators targeting the receptor’s intracellular domain. Therapeutic strategies targeting PAR1 have predominantly employed oral or intravenous routes to mitigate thrombosis and treat cardiovascular diseases. However, emerging research underlining the role of PAR1 activation in promoting wound healing,^60^ inflammatory responses^61,62^ and tumor invasion^63,64^ suggests novel therapeutic strategies, including the development of PAR1 agonists as topical agents for wound management, antagonists to alleviate itch,^62^ and a deeper investigation into the anti-tumor potential of PAR1-targeted therapies. Further research is required to investigate and broaden the therapeutic potential of PAR1 modulation in the aforementioned contexts.

## Materials and methods

### Construct design

An HA signal peptide was fused to the N-terminus of WT PAR1 (residues 42–397). A 15-amino acid linker (GSSGGGGSGGGGSSG) was inserted between the C-terminus of PAR1 and a LgBiT tag, followed by a TEV protease cleavage site and a double MBP tag at the C-terminus. The resulting construct is cloned into pFastBac vector (Invitrogen). The engineered G_q_ is derived from mini-G_s/q_, two dominant negative mutations were introduced to decrease the binding ability of nucleotide; the N-terminal 1–18 amino acids and α-helical domain of human Gα_i_ replaced the corresponding sequences of Gα_q_ to accommodate the binding of scFv16.^27^ Gα_i_ containing two dominant-negative mutations (G203A and A326S)^28^ was used to obtain a stable GPCR–G_i_ complex. Rat Gβ1 was cloned with an N-terminal His6 tag and a C-terminal 15-amino acid linker, followed by a HiBiT tag. The antibody scFv16 was cloned with an N-terminal GP67 signal peptide. All the G protein components, including bovine Gγ2 and the antibody scFv16, were cloned into the pFastBac vector, respectively.

### Expression and purification of PAR1–G protein complexes

Bac-to-Bac^TM^ Baculovirus Expression System (Invitrogen) was used to generate recombinant viruses of PAR1, Gα_q_, Gα_i_, Gβ1, Gγ2, and scFv16 in Sf9 insect cells. Sf9 cells at a density of 3 × 10^6^ cells/mL were transfected with the viruses of PAR1, Gα (Gα_i_ or Gα_q_), Gβ1, Gγ2, and scFv16 at a ratio of 1:1:1:1:1 and cultured for 48 h at 27 °C. After infection, cells were collected by centrifugation, and the cell pellets were stored at −80 °C.

The cell pellets were suspended and lysed by homogenization in 20 mM HEPES, pH 7.5, 100 mM NaCl, 5 mM CaCl_2_, 10 mM MgCl_2_, and 100 μM TCEP supplemented with Protease Inhibitor Cocktail (Bimake). PAR1–G protein complexes were assembled at room temperature for 1.5 h by the addition of 10 μM GB83 (homemade) and 30 mU/mL apyrase (sigma). Then the lysate was solubilized by addition of 0.5% (w/v) *n*-Dodecyl-β-d-Maltopyranoside (DDM, Anatrace), 0.1% (w/v) cholesteryl hemisuccinate TRIS salt (CHS, Anatrace) for 3 h at 4 °C. The supernatant was separated by centrifugation at 30,000 rpm for 30 min and incubated with dextrin resin (Dextrin Beads 6FF, SMART Lifesciences) at 4 °C for 3 h. After incubation, the resin was loaded onto a gravity flow column and washed with 20 column volumes of buffer consisting of 20 mM HEPES, pH 7.5, 100 mM NaCl, 100 μM TCEP, 10 μM GB83, 0.05% (w/v) lauryl maltose neopentylglycol (LMNG, Anatrace), and 0.01% (w/v) CHS. After being washed, the resin was resuspended with 5 column volumes of buffer containing 20 mM HEPES, pH 7.5, 100 mM NaCl, 100 μM TCEP, 10 μM GB83, 0.03% (w/v) LMNG, 0.01% (w/v) CHS, and 0.005% (w/v) glycol-diosgenin (GDN, Anatrace) supplemented with TEV protease (homemade) to remove the C-terminal double MBP tag and incubated at 4 °C overnight. The flow-through was collected and concentrated, then loaded onto a Superdex 200 increase 10/300 column (GE Healthcare) or Superose 6 increase 10/300 column (GE Healthcare) preequilibrated with running buffer containing 20 mM HEPES, pH 7.5, 100 mM NaCl, 0.00075% (w/v) LMNG, 0.00025% (w/v) GDN, 0.00015% (w/v) CHS, and 10 μM GB83. The fractions for the monomeric complex were collected and concentrated for EM experiments.

### Cryo-EM grid preparation and data collection

For the preparation of cryo-EM grids, 2.6 μL of purified complexes at ~10 mg/mL for the TA–PAR1–G_q_ complex and ~5.0 mg/mL for the TA–PAR1–G_i_ complex were applied to glow-discharged holey carbon grids (Quantifoil R1.2/1.3), respectively. The grids were vitrified in liquid ethane by Vitrobot Mark IV (FEI). Frozen grids were transferred to liquid nitrogen and stored until data collection. Cryo-EM imaging was performed on a Titan Krios (FEI) at 300 kV using a Gatan K3 summit direct electron detector with a Gatan energy filter with a slit width of 20 eV at the Center of Cryo-Electron Microscopy Research Center, Shanghai Institute of Materia Medica, Chinese Academy of Sciences (Shanghai, China). Micrographs were recorded by SerialEM software in super-resolution mode at a magnified physical pixel size of 1.071 Å, with defocus values ranging from −0.5 μm to −3.0 μm. The total exposure time was set to 3 s with intermediate frames recorded every 0.083 s, resulting in an accumulated dose of ~70 electrons per Å^2^ and a total of 36 frames per movie stack. A total of 5048 and 6097 movies were collected for the TA–PAR1–G_q_ complex and the TA–PAR1–G_i_ complex, respectively.

### Cryo-EM data processing

The image stacks were aligned to correct for drift and beam-induced motion using MotionCor2.1.^65^ Contrast transfer function (CTF) parameter of each micrograph was estimated by CTFFIND4.1.^66^ Cryo-EM data processing was performed using RELION-3.1.0.^67^ For the two complexes, particle selections for 2D and 3D classifications were performed on a binned dataset with a pixel size of 1.071 Å.

For the TA–PAR1–G_q_ complex, the micrographs containing carbon area and severe ice contamination were discarded; auto-picking of the remained micrographs yielded 3,944,919 particles, which were subjected to 3 rounds of reference-free 2D classification to discard poorly defined particles. An initial model generated from the 2D classification-selected particles by RELION was used as a reference for 3D classifications. The particles were subjected to 2 rounds of 3D classification for the whole complex, a round of 3D classification with a mask on the receptor, and another round of 3D classification focused on the complex, resulting in one well-defined subset with 723,778 particles. The selected subset was subjected to 3D auto-refine, CTF refinement, and Bayesian polishing. The final refinement generated a map with an indicated global resolution of 3.0 Å at a Fourier shell correlation of 0.143.

For the TA–PAR1–G_i_ complex, the micrographs containing carbon area and severe ice contamination were discarded; auto-picking of the remained micrographs yielded 5,979,500 particles which were subjected to 3 rounds of reference-free 2D classification to discard poorly defined particles. An initial model generated from 2D classification selected particles by RELION was used as a reference for 3D classifications. The particles were subjected to 3 rounds of 3D classification for the whole complex, a round of 3D classification with a mask on the receptor, and another round of 3D classification focused on the complex, resulting in one well-defined subset with 162,724 particles. The selected subset was subjected to 3D auto-refine, CTF refinement, and Bayesian polishing. The final refinement generated a map with an indicated global resolution of 3.2 Å at a Fourier shell correlation of 0.143. Local resolution for both maps was determined using Resmap^68^ with half maps as input.

### Model building and refinement

For the TA–PAR1–G_q_ complex, the initial PAR1 model was adopted from the crystal structure of human PAR1 in complex with vorapaxar (PDB: 3VW7)^10^ and the G_q_ protein model was adopted from the cryo-EM structure of the 5-HT_2A_–G_q_ protein complex (PDB: 6WHA).^69^ The models were docked into the density map using Chimera and manually adjusted and rebuilt in COOT^70^ and ISOLDE.^71^ Real space refinement was performed using Phenix^72^ programs, and the validation of model statistics was done using MolProbity.^73^ For the TA–PAR1–G_i_ complex, the refined PAR1 model in the TA–PAR1–G_q_ complex was used, and the G_i_ protein model was adopted from the cryo-EM structure of FPR2–G_i_ protein complex (PDB: 6OMM).^74^ The adopted model was built in the same way as the former complex. The final refinement statistics are provided in Supplementary information, Table S1. Structural figures were prepared with Chimera,^75^ ChimeraX^76^ and PyMOL.^77^

### NanoBiT-G protein dissociation assay

The NanoBiT assay for the measurement of G protein activation was performed as previously described.^78^ In brief, HEK293T cells were plated in each well of a six-well plate at a concentration of 0.3 million/mL (3 mL per well). For the G_q_ protein dissociation assay, HEK293T cells were co-transfected with a plasmid mixture of 200 ng Gα_q_-LgBiT, 500 ng SmBiT-Gγ, 500 ng Gβ, 600 ng PAR1 receptor (1–425 amino acids) and 200 ng Ric8a by Lipofectamine 2000 (Thermo Fisher Scientific) in 200 μL of Opti-MEM (Gibco). For G_i_ protein dissociation assay, the mixture of plasmid transfection contains 200 ng Gα_i_-LgBiT, 500 ng SmBiT-Gγ, 500 ng Gβ and 600 ng PAR1 receptor (1–425 amino acids). After 1 day of transfection, cells in the six-well plate were digested and resuspended in complete medium DMEM (10% FBS, 1% antibiotic) and plated in a 96-well flat-bottomed white microplate (WHB). After 18–24 h, the cells were washed twice with D-PBS to remove cell medium and incubated in the assay buffer (HBSS containing 0.01% BSA and 5 mM HEPES, pH 7.5) containing 10 μM coelenterazine 400a (Maokangbio) at a volume of 45 μL per well for 45 min at room temperature. Baseline luminescence was measured using a luminescent microplate reader (Spark Multimode Microplate Reader, TECAN). After adding 5 μL TA peptide (amino acid sequence: SFLLRN) (10×, gradient diluted in the assay buffer), the plate was measured in a luminescent microplate reader immediately. The ligand-induced signal ratio was normalized to the baseline luminescence, and fold-change signals over vehicle treatment were used to show the G protein dissociation response. Finally, the ligand-induced signal ratio was normalized to 100% of the maximal response of WT PAR1 using a sigmoidal dose response in GraphPad Prism. For G_q_ protein dissociation induced by thrombin, the methods are generally the same, expect for using 2 U/well thrombin (Yeasen) instead of the TA peptide to activate the receptor.

### Cell-surface enzyme-linked immunosorbent assay (ELISA)

Cell-surface expression levels of PAR1 and the mutants were determined by ELISA. The transfected cells were washed with 1× PBS and fixed with 45 μL of 4% paraformaldehyde for 10 min. Following fixation, cells were blocked with blocking buffer (1% (w/v) BSA/PBS) for 1 h at room temperature. Afterward, cells were incubated with a 1:5000 dilution of HRP-conjugated mouse anti-HA-Tag mAb (Roche) in a blocking buffer for another 0.5 h at room temperature. Then, wells were washed three times with blocking buffer and three times with 1× PBS in order. Finally, antibody binding was detected using 80 μL/well diluent SuperSignal Elisa Femto Maximum Sensitivity Substrate (Thermo Fisher Scientific). The plate was measured for luminescence (Spark Multimode Microplate Reader, TECAN). Finally, the data were normalized to 100% of the WT PAR1 using GraphPad Prism.

### Statistical analysis

Statistical analyses were performed on at least three individual datasets and analyzed using the GraphPad Prism software. Bars represent differences in the calculated agonist potency (ΔpEC_50_ = pEC_50_ of mutant – pEC_50_ of WT) and efficacy (Δefficacy = efficacy of mutant – efficacy of WT) for each mutant relative to the WT receptor. Data shown were means ± SEM from at least three independent experiments performed in technical triplicate. NS, *P* ≥ 0.05, *P < 0.05, **P < 0.01, ***P < 0.001, and ****P < 0.0001 by one-way ANOVA followed by Fisher’s LSD multiple comparisons test compared with WT. For dose-response experiments, data were normalized and analyzed using nonlinear curve fitting for the log (agonist) vs response (three parameters) curves. For the comparison between G_q_–protein dissociation induced by TA peptide and thrombin, data were analyzed using a two-way ANOVA followed by multiple comparisons.

## Supplementary information

Supplementary information, Fig. S1

Supplementary information, Fig. S2

Supplementary information, Fig. S3

Supplementary information, Fig. S4

Supplementary information, Fig. S5

Supplementary information, Fig. S6

Supplementary information, Fig. S7

Supplementary information, Fig. S8

Supplementary information, Fig. S9

Supplementary information, Table S1

Supplementary information, Table S2

Supplementary information, Table S3

Supplementary information, Table S4

Supplementary information, Table S5

Supplementary information, Table S6

Supplementary information, Table S7

## Data Availability

The cryo-EM maps have been deposited in the Electron Microscopy Data Bank (EMDB) under accession codes EMD-38538 (PAR1–G_q_), and EMD-38539 (PAR1–G_i_). The coordinates have been deposited in the Protein Data Bank (PDB) under accession codes 8XOR (PAR1–G_q_), and 8XOS (PAR1–G_i_).
