# Supplementary material for: Structural basis of tethered agonism and G protein coupling of protease-activated receptors
Source: Cell Res. 2024 Jul 12;34(10):725–34. doi: 10.1038/s41422-024-00997-2 (PMC11443083; doi:10.1038/s41422-024-00997-2)
Supplement: Supplementary file 1 — Supplementary information, Fig. S1 [file 41422_2024_997_MOESM1_ESM.pdf]

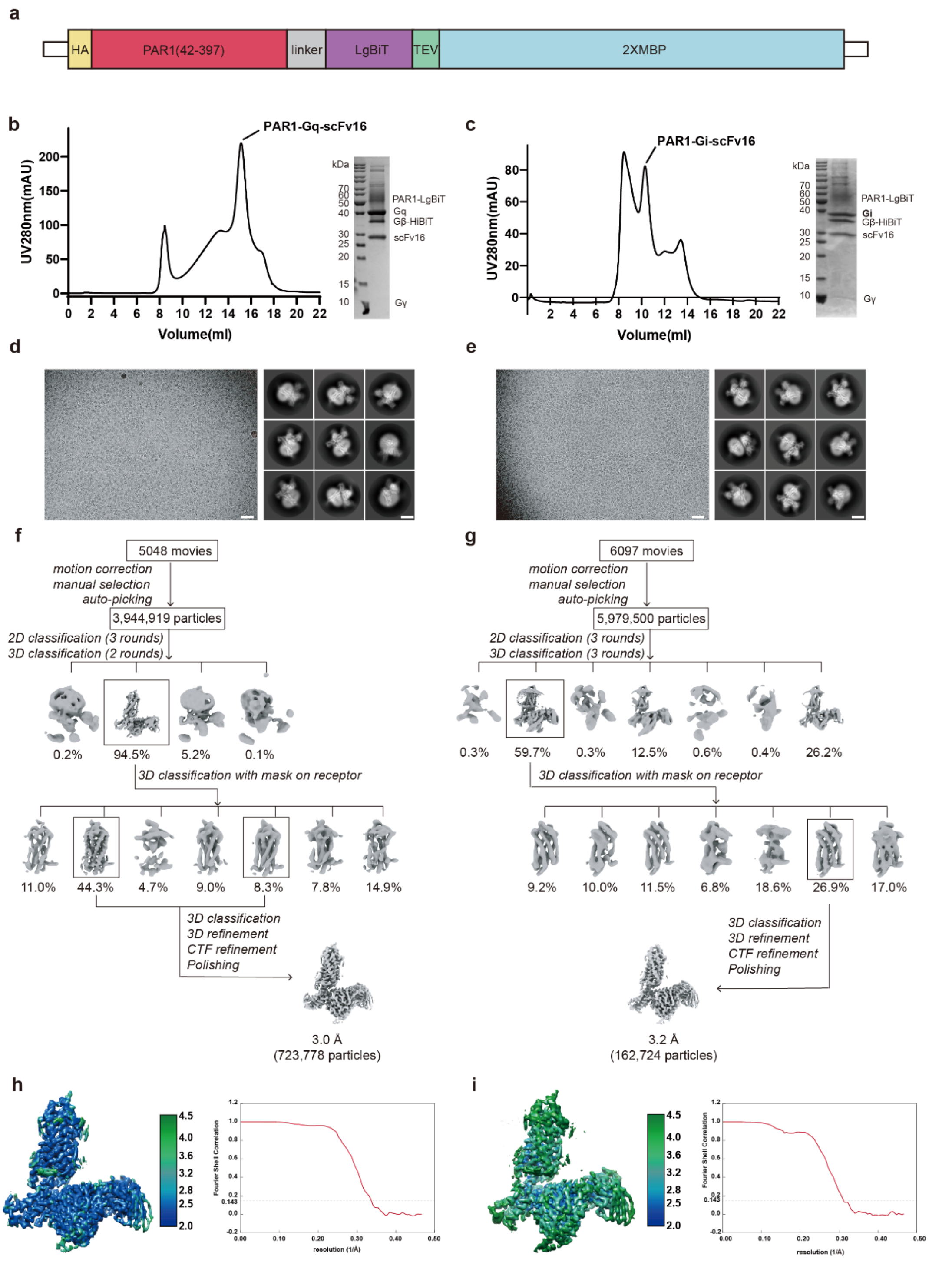

Supplementary information, Fig. S1 Sample preparation and representative data process of TA-PAR1-G protein complexes. a, Schematic diagram of the PAR1 construct used in this study. **b,** Size-exclusion chromatography on a Superose 6 Increase 10/300GL and SDS-PAGE analysis of the TA-PAR1-G_q-scFv16 complex. **c,** Size-exclusion chromatography on a Superdex

200 increase 10/300 column and SDS-PAGE analysis of the TA-PAR1-G_i-scFv16 complex. **d, e,** Cryo-EM micrographs of the TA-PAR1-G_q-scFv16 complex (**d**) and TA-PAR1-G_i-scFv16 complex (**e**) (scale bar: 30 nm) and 2D class averages (scale bar: 5 nm). **f, g,** Flow chart of the cryo-EM data processing and 3D reconstruction workflow for the TA-PAR1-G_q-scFv16 complex (**f**) and TA-PAR1-G_i-scFv16 complex (**g**). **h, i,** Cryo-EM maps, and "Gold-standard" FSC curves corresponding to the TA-PAR1-G_q-scFv16 complex (**h**) and TA-PAR1-G_i-scFv16 complex (**i**) colored by local resolution (Å).
