# Supplementary material for: Structural basis of tethered agonism and G protein coupling of protease-activated receptors
Source: Cell Res. 2024 Jul 12;34(10):725–34. doi: 10.1038/s41422-024-00997-2 (PMC11443083; doi:10.1038/s41422-024-00997-2)
Supplement: Supplementary file 2 — Supplementary information, Fig. S2 [file 41422_2024_997_MOESM2_ESM.pdf]

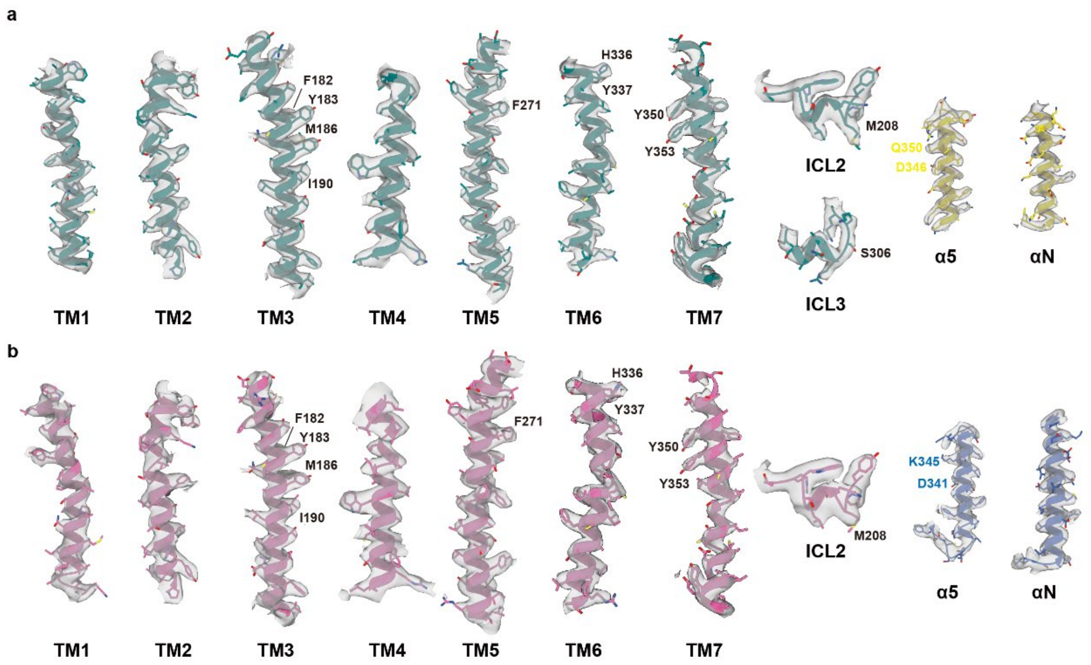

Supplementary information, Fig. S2. Representative density map of TA-PAR1-G protein complexes. a, Cryo-EM density maps and the models of PAR1-G_q-scFv16 complex for all transmembrane helices, ICL2, ICL3, helix 5 of $G\alpha_q$ and GαN of $G\alpha_q$. **b,** Cryo-EM density maps and the models of PAR1-G_i-scFv16 complex for all transmembrane helices, ICL2, helix 5 of $G\alpha_i$ and GαN of $G\alpha_i$.
