# Supplementary material for: Structural basis of tethered agonism and G protein coupling of protease-activated receptors
Source: Cell Res. 2024 Jul 12;34(10):725–34. doi: 10.1038/s41422-024-00997-2 (PMC11443083; doi:10.1038/s41422-024-00997-2)
Supplement: Supplementary file 3 — Supplementary information, Fig. S3 [file 41422_2024_997_MOESM3_ESM.pdf]

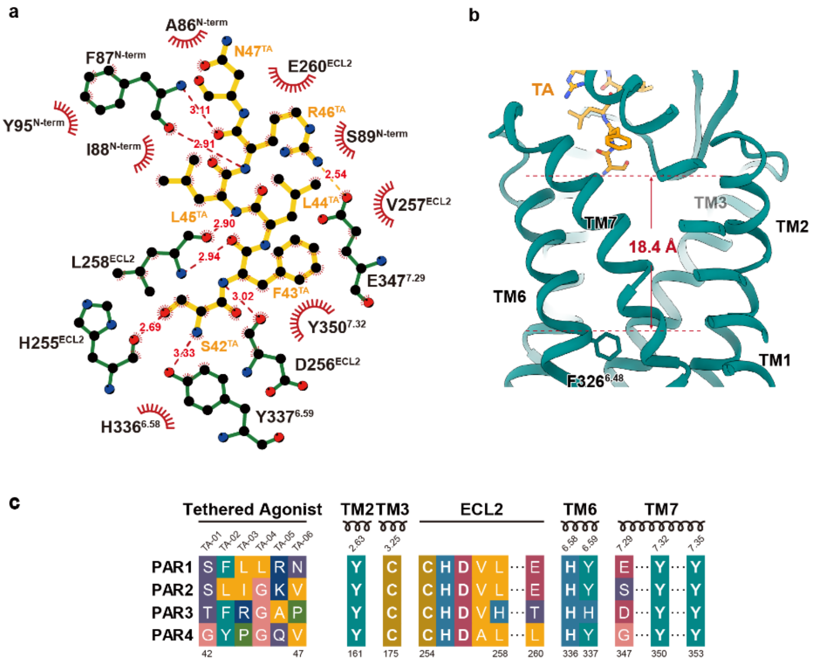

Supplementary information, Fig. S3. Ligand binding mode of PAR1. a, Pattern diagram of the interactions between the TA peptide and receptor. Hydrogen bonds are depicted as red dashed lines. Salt bridge is depicted as orange dashed lines. **b,** The distance from the TA to $F^{6.48}$. **c,** Sequences alignment of amino acids in the ligand binding pocket in PAR1 with the PAR family. Coils represent α-helices. The positions that share fully conserved residues were highlighted in bold.
