# Supplementary material for: Structural basis of tethered agonism and G protein coupling of protease-activated receptors
Source: Cell Res. 2024 Jul 12;34(10):725–34. doi: 10.1038/s41422-024-00997-2 (PMC11443083; doi:10.1038/s41422-024-00997-2)
Supplement: Supplementary file 4 — Supplementary information, Fig. S4 [file 41422_2024_997_MOESM4_ESM.pdf]

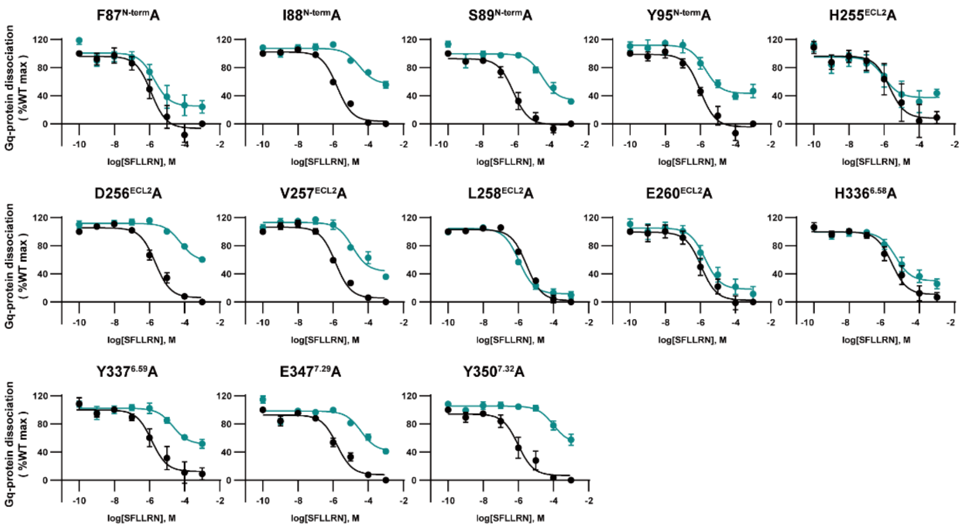

Supplementary information, Fig. S4. Effects of mutations in the ligand binding pocket of PAR1. Mutant study to assess TA response by the NanoBiT-G_q dissociation assay. The data are presented as mean values ± SEM from at least three independent experiments performed in technical triplicate. The data were normalized according to the maximal response of WT PAR1. For details of the ΔpEC50, Emax and error values, see Supplementary information, Table S3.

.
