# Supplementary material for: Structural basis of tethered agonism and G protein coupling of protease-activated receptors
Source: Cell Res. 2024 Jul 12;34(10):725–34. doi: 10.1038/s41422-024-00997-2 (PMC11443083; doi:10.1038/s41422-024-00997-2)
Supplement: Supplementary file 5 — Supplementary information, Fig. S5 [file 41422_2024_997_MOESM5_ESM.pdf]

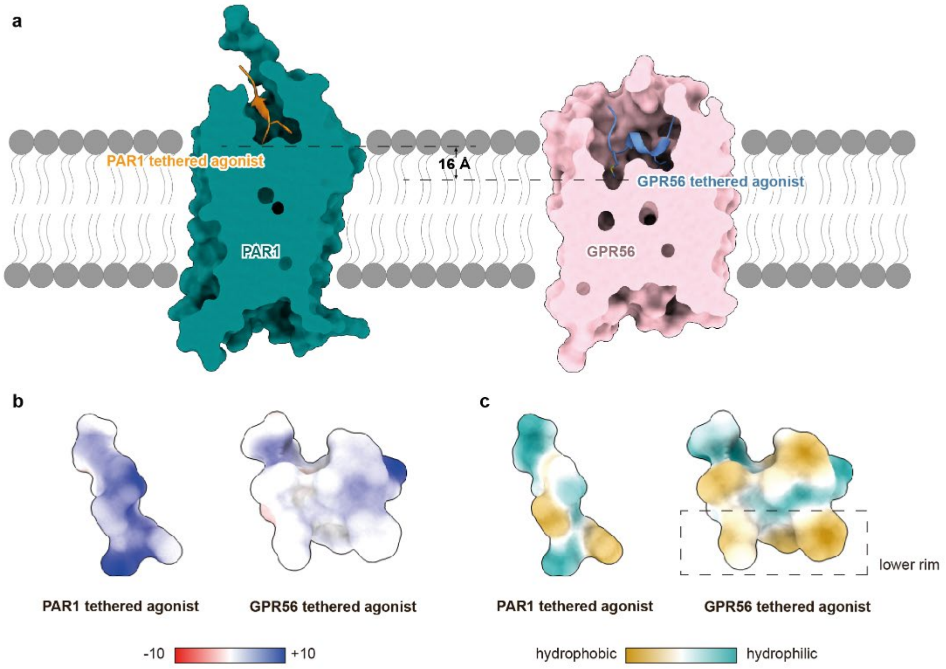

Supplementary information, Fig. S5. Structure comparison of ligand binding pockets in tethered agonist-activated GPCRs. a, Surface cut-away views of tethered agonist-binding pockets of PAR1 (teal) and GPR56 (pink) (PDB code: 7SF8[39]). Receptors were shown as surface, tethered agonists were shown as ribbon. **b,** Surface representation of the tethered agonists of PAR1 and GPR56 colored by electrostatic potential, red (−10 kT/e), blue (+10 kT/e), and white (neutral). **c,** Surface representation of the tethered agonists of PAR1 and GPR56 is colored by residue hydrophobicity.
