# Supplementary material for: Structural basis of tethered agonism and G protein coupling of protease-activated receptors
Source: Cell Res. 2024 Jul 12;34(10):725–34. doi: 10.1038/s41422-024-00997-2 (PMC11443083; doi:10.1038/s41422-024-00997-2)
Supplement: Supplementary file 6 — Supplementary information, Fig. S6 [file 41422_2024_997_MOESM6_ESM.pdf]

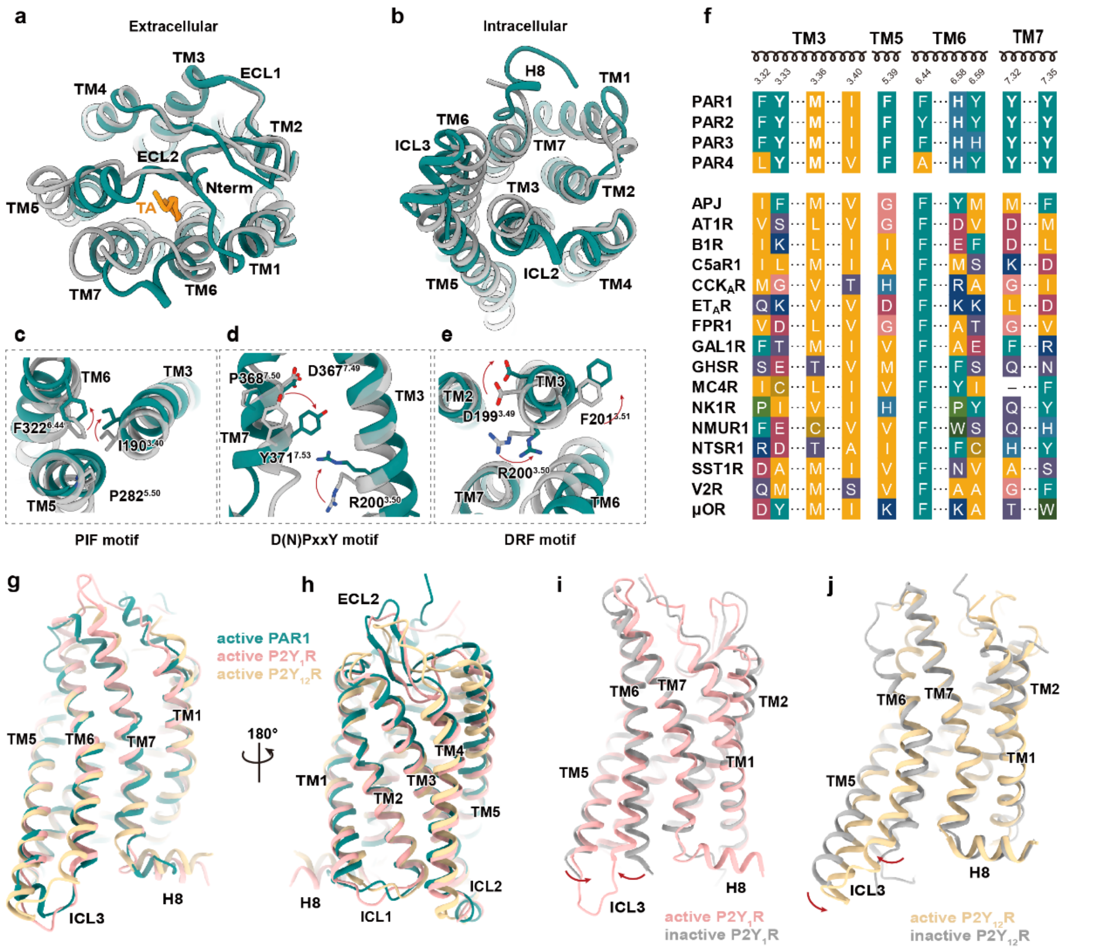

Supplementary information, Fig. S6. Comparison among structural motifs in the active and inactive states of PAR1 and purinergic receptors. a, b, Superposition of TA bound PAR1 structure (teal) with inactive PAR1 structure (light gray) (PDB code: 3VW7) viewed from the extracellular space (**a**) and intracellular space (**b**). The TA was shown as yellow sticks. **c, d, e,** Conformation changes of $P^{5.50}I^{3.40}F^{6.44}$ motif, $D^{7.49}P^{7.50}$ $xxY^{7.53}$ motif, and $D^{3.49}R^{3.50}F^{3.51}$ motif. The movements of TM2, TM3, TM6, and TM7 in the active PAR1 compared with the inactive PAR1 are shown with red arrow. **f,** Sequences alignment of class A peptide receptors. α-helices are shown as coils. The positions which share fully conserved residues in the PAR subfamily were highlighted in bold. **g, h,** The superposition of active PAR1, $P2Y_1R$ (pink) (PDB: 7XXH), and $P2Y_{12}R$ (yellow) (PDB: 7XXI). **i,** Superposition of active $P2Y_1R$ (pink) with inactive $P2Y_1R$ (gray). **j,** Superposition of active $P2Y_{12}R$ (yellow) with inactive $P2Y_{12}R$ (gray).
