# Supplementary material for: Structural basis of tethered agonism and G protein coupling of protease-activated receptors
Source: Cell Res. 2024 Jul 12;34(10):725–34. doi: 10.1038/s41422-024-00997-2 (PMC11443083; doi:10.1038/s41422-024-00997-2)
Supplement: Supplementary file 7 — Supplementary information, Fig. S7 [file 41422_2024_997_MOESM7_ESM.pdf]

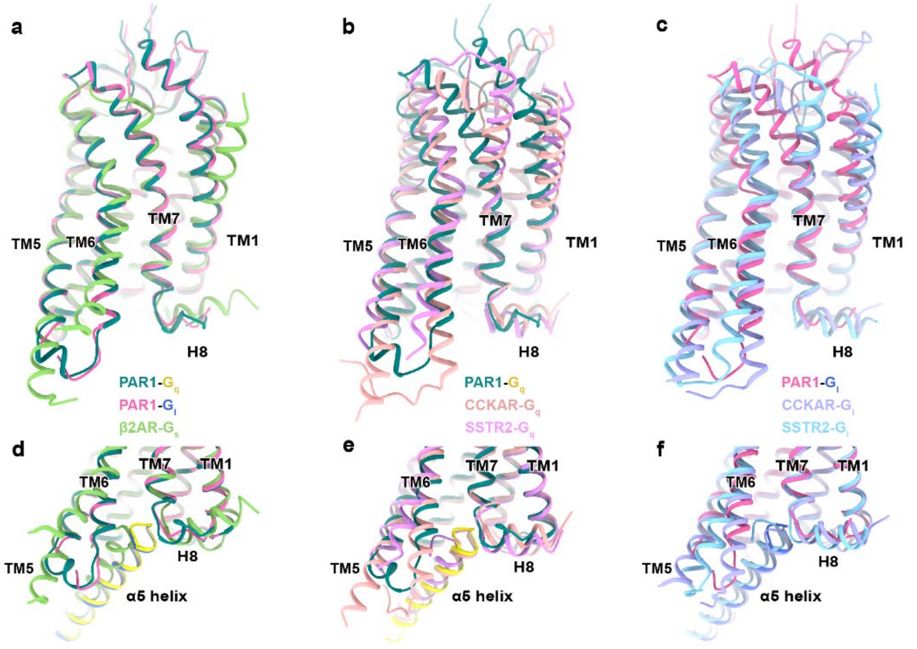

Supplementary information, Fig. S7. Comparison of the active structures of PAR1 and other Class A GPCRs. a, Side views of the receptors in PAR1-G_q (teal) and PAR1-G_i (megenta) complexes compared to the receptor in β_2AR-G_s complex (green) (PDB code: 3SN6). **b,** Side views of the receptors in PAR1-G_q complex compared to the receptor in CCK_AR-G_q (pink) (PDB code: 7EZM) and SSTR2-G_q complexes (salmon) (PDB code: 7Y27). **c,** Side views of the receptors in PAR1-G_i complex compared to the receptor in CCK_AR-G_i (purple) (PDB code: 7EZH) and SSTR2-G_i complexes (blue) (PDB code: 7WIC). **d-f,** Close-up comparisons of the G protein binding pocket among the previously mentioned structures.
