# Supplementary material for: Structural basis of tethered agonism and G protein coupling of protease-activated receptors
Source: Cell Res. 2024 Jul 12;34(10):725–34. doi: 10.1038/s41422-024-00997-2 (PMC11443083; doi:10.1038/s41422-024-00997-2)
Supplement: Supplementary file 8 — Supplementary information, Fig. S8 [file 41422_2024_997_MOESM8_ESM.pdf]

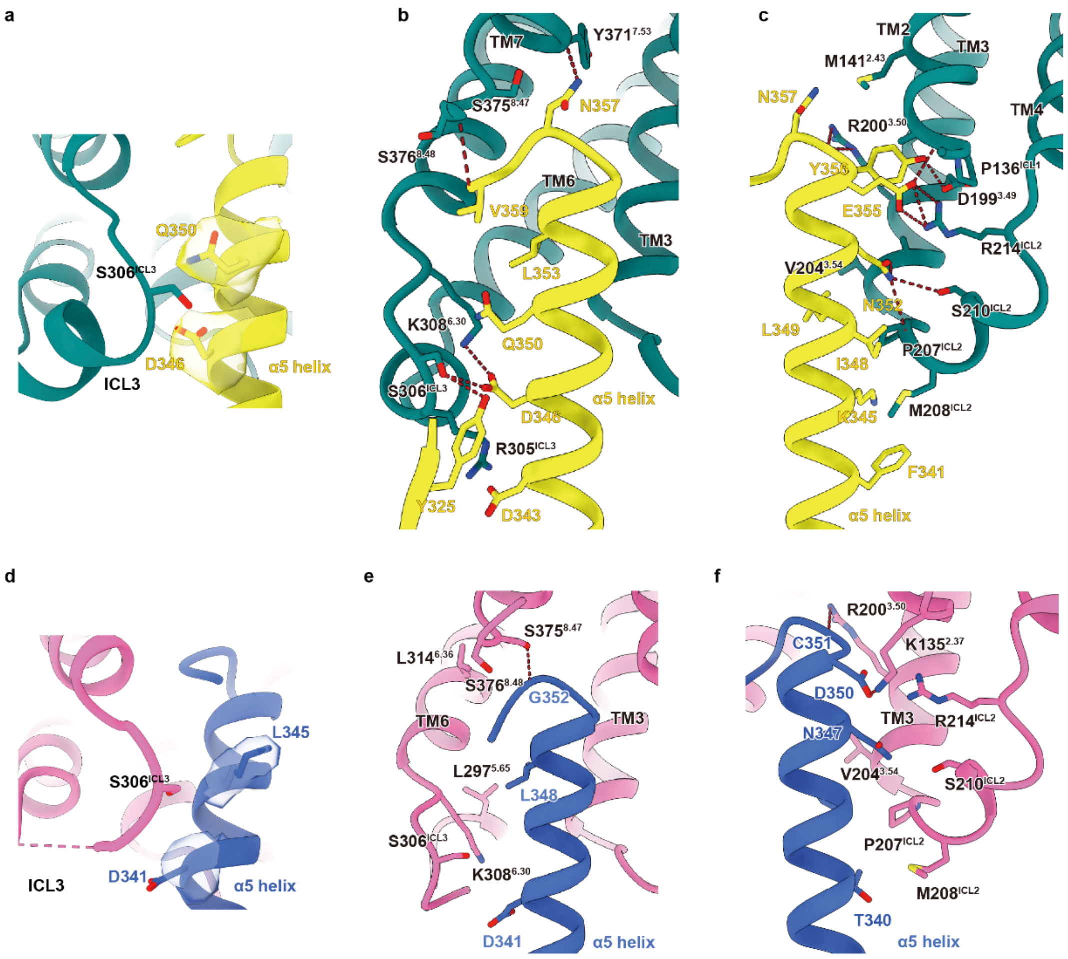

Supplementary information, Fig. S8. Structural comparison of Gα_q protein with Gα_i protein coupling interface. a, Close-up view of Cryo-EM density maps of the key amino acids in the α5 helix of the Gα_q subunit (yellow) interacting with ICL3. **b, c,** Close-up view of the interactions between the α5 helix of the Gα_q subunit (yellow) and amino acids in the cytoplasmic cavity of PAR1 (teal). **d,** Close-up view of Cryo-EM density maps of the key amino acids in α5 helix of the Gα_i subunit (blue) interacting with ICL3. **e, f,** Close-up view of the interactions between the α5 helix of the Gα_i subunit (blue) and amino acids in the cytoplasmic cavity of PAR1 (pink). Amino acids involved in binding are shown as sticks. Polar interactions are shown as red dashed lines.
