# Supplementary material for: Structural basis of tethered agonism and G protein coupling of protease-activated receptors
Source: Cell Res. 2024 Jul 12;34(10):725–34. doi: 10.1038/s41422-024-00997-2 (PMC11443083; doi:10.1038/s41422-024-00997-2)
Supplement: Supplementary file 9 — Supplementary information, Fig. S9 [file 41422_2024_997_MOESM9_ESM.pdf]

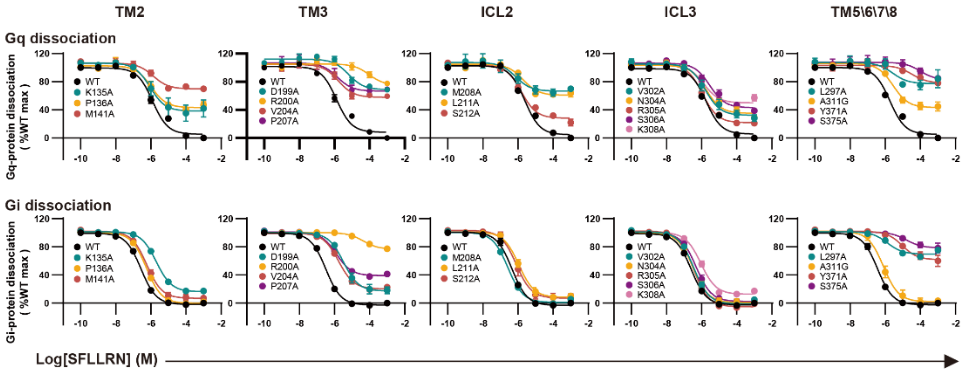

Supplementary information, Fig. S9. Mutant studies assessing G protein coupling to PAR1.

Dose-response curves of NanoBiT-G_q dissociation assay and NanoBiT-G_i dissociation assay of WT and the mutant PAR1 stimulated by externally added TA peptide. The data are presented as mean values ± SEM from at least three independent experiments performed in technical triplicate. The data were normalized according to the maximal response of WT PAR1. For details of the ΔpEC50, Emax and error values, see Supplementary information, Tables S6, S7.
