# Supplementary material for: Structural basis of tethered agonism and G protein coupling of protease-activated receptors
Source: Cell Res. 2024 Jul 12;34(10):725–34. doi: 10.1038/s41422-024-00997-2 (PMC11443083; doi:10.1038/s41422-024-00997-2)
Supplement: Supplementary file 10 — Supplementary information, Table S1 [file 41422_2024_997_MOESM10_ESM.pdf]

Table S1. Cryo-EM data collection, model refinement and validation statistics.

	TA-PAR1-G_q-scFv16	TA-PAR1-G_i-scFv16
**Data collection and processing**		
Magnification	81,000	81,000
Voltage (kV)	300	300
Electron exposure ($e^-/Å^2$)	70	70
Defocus range (μm)	1.0 to 3.0	1.0 to 3.0
Pixel size (Å)	1.071	1.071
Symmetry imposed	C1	C1
Initial particle projections (no.)	3,944,919	5,979,500
Final particle projections (no.)	723,778	162,724
Map resolution (Å)	3.0	3.2
FSC threshold	0.143	0.143
Map resolution range (Å)	2.5-5.0	2.5-5.0
**Refinement**		
Initial model used (PDB accession number)	3VW7 & 6WHA	3VW7 & 6OMM
Model Resolution (Å)	3.3	3.5
FSC threshold	0.5	0.5
Map sharpening method	Relion	Relion
**Model Composition**		
Non-hydrogen atoms	8980	8920
Protein residues	1160	1136
Lipid	6	2
B factors ($Å^2$)		
Protein (min/max/mean)	30.00/290.12/124.80	30.00/228.06/108.92
Lipid (min/max/mean)	132.19/146.11/140.50	113.82/114.83/114.33
**RMSD**		
Bound lengths (Å)	0.006	0.018
Bound angles (°)	1.197	1.209
**Validation**		
MolProbity score	1.58	1.66
Clash score	5.35	6.19
Rotamer outliers (%)	0.43	0.52
**Ramachandran Plot**		
Favored (%)	95.80	95.43
Allowed (%)	4.20	4.57
Disallowed (%)	0	0
**Accession number**		
EMDB	EMD-38538	EMD-38539
PDB	8XOR	8XOS

FSC, Fourier correlation; EMDB, Electron Microscopy Data Bank; PDB, Protein Data Bank; RMSD, root-mean-square deviation.
