# Supplementary material for: Structural basis of tethered agonism and G protein coupling of protease-activated receptors
Source: Cell Res. 2024 Jul 12;34(10):725–34. doi: 10.1038/s41422-024-00997-2 (PMC11443083; doi:10.1038/s41422-024-00997-2)
Supplement: Supplementary file 11 — Supplementary information, Table S2 [file 41422_2024_997_MOESM11_ESM.pdf]

Table S2. Intramolecular interactions between the TA and PAR1 in TA-PAR1-G_q complex.

Amino acid in the TA	Interaction partner	Interaction type	Distance (Å)
S42	$H255^{ECL2}$	Hydrogen bond	2.69
	$D256^{ECL2}$	Polar interaction	3.23
	$V257^{ECL2}$	Van der Waals force	3.69
	$L258^{ECL2}$	Van der Waals force	3.33
	$H336^{6.58}$	Polar interaction	3.41
	$Y337^{6.59}$	Hydrogen bond	3.33
	$Y350^{7.32}$	Van der Waals force	3.41
F43	$Y95^{N\text{-}term}$	Hydrophobic interaction	3.96
	$D256^{ECL2}$	Hydrogen bond	3.02
	$V257^{ECL2}$	Hydrophobic interaction	3.48
	$L258^{ECL2}$	Hydrogen bond	2.94
	$E347^{7.29}$	Van der Waals force	3.50
	$Y350^{7.32}$	π-π stacking	3.67
L44	$I88^{N\text{-}term}$	Hydrophobic interaction	3.74
	$L258^{ECL2}$	Hydrophobic interaction	3.59
	$E260^{ECL2}$	Van der Waals force	3.80
L45	$V257^{ECL2}$	Hydrophobic interaction	3.59
	$L258^{ECL2}$	Hydrogen bond	2.90
	$E260^{ECL2}$	Van der Waals force	3.18
R46	$A86^{N\text{-}term}$	Van der Waals force	3.44
	$F87^{N\text{-}term}$	Hydrogen bond	2.91
	$S89^{N\text{-}term}$	Polar interaction	3.71
	$E260^{ECL2}$	Polar interaction	3.48
	$E347^{7.29}$	Salt bridge	2.54
