# Supplementary material for: Structural basis of tethered agonism and G protein coupling of protease-activated receptors
Source: Cell Res. 2024 Jul 12;34(10):725–34. doi: 10.1038/s41422-024-00997-2 (PMC11443083; doi:10.1038/s41422-024-00997-2)
Supplement: Supplementary file 12 — Supplementary information, Table S3 [file 41422_2024_997_MOESM12_ESM.pdf]

Table S3. SFLLRN–induced G_q dissociation of WT and mutant PAR1.

	EC50 (μM) ±SEM[a]	pEC50±SEM[a]	ΔpEC50±SEM[a]	Efficacy±SEM[a,b] (%WT)	ΔEfficacy±SEM[a,b] (%WT)	Sample size	Expression (%WT)
WT	2.767±1.14	5.72±0.11	0	100	0	5	100
F87$^{N\text{-}term}$A	4.70±3.11^{NS}	5.58±0.25^{NS}	-0.22±0.06^{NS}	73.85±7.10**	-26.15±7.10**	4	70.24±14.45
I88$^{N\text{-}term}$A	37.29±10.63*	4.47±0.16****	-1.30±0.17****	52.41±3.00****	-47.59±3.00****	3	70.99±5.031
S89$^{N\text{-}term}$A	35.8±11.13*	4.51±0.18****	-1.74±0.09****	70.79±4.41**	-29.21±4.41**	3	94.15±9.11
Y95$^{N\text{-}term}$A	1.47±0.43^{NS}	5.95±0.24^{NS}	-0.23±0.13^{NS}	64.07±0.87****	-35.93±0.87****	4	71.94±16.49
H255^{ECL2}A	3.80±3.31^{NS}	5.89±0.47^{NS}	0.11±0.08^{NS}	61.87±5.52****	-38.13±5.52****	3	62.56±9.14
D256^{ECL2}A	47.42±5.37***	4.33±0.05****	-1.53±0.07****	56.63±6.73****	-43.37±6.73****	3	63.97±7.12
V257^{ECL2}A	13.07±0.09^{NS}	4.89±0.03*	-0.98±0.06****	69.00±1.18***	-31.00±1.18***	3	67.83±12.39
L258^{ECL2}A	1.11±0.01^{NS}	5.96±0.01^{NS}	0.43±0.02^{NS}	91.88±2.62^{NS}	-8.12±2.62^{NS}	3	146.00±12.31
E260^{ECL2}A	1.98±0.43^{NS}	5.73±0.11^{NS}	-0.21±0.05^{NS}	89.82±6.33^{NS}	-10.18±6.33^{NS}	3	42.96±7.50
H336$^{6.58}$A	1.67±0.61^{NS}	5.87±0.22^{NS}	-0.34±0.12^{NS}	73.05±6.27**	-26.95±6.27**	4	88.83±17.20
Y337$^{6.59}$A	19.90±0.82^{NS}	4.88±0.27**	-1.19±0.23****	54.72±4.06****	-45.28±4.06****	3	54.56±11.01
E347$^{7.29}$A	48.53±13.85***	4.33±0.09****	-1.22±0.16****	55.68±8.07****	-44.32±8.07****	5	68.37±8.73
Y350$^{7.32}$A	111.50±27.73****	3.98±0.11****	-2.20±0.23****	68.58±1.49***	-31.42±1.49***	3	66.34±15.42

[a]NanoBiT results of G_q protein dissociation for PAR1 (WT and mutant) were normalized to the maximal response of wild-type PAR1. The data are presented as means ± SEM from at least three independent experiments performed in technical triplicate. $^{NS}P > 0.05$, $^{*}P < 0.05$, $^{**}P < 0.01$, $^{***}P < 0.001$ and $^{****}P < 0.0001$ by one-way ANOVA followed by Fisher's LSD multiple comparisons test compared with WT PAR1.

[b]The efficacy is defined as the window between the maximal response (E_{max}) and the vehicle (no agonist).
