# Supplementary material for: Structural basis of tethered agonism and G protein coupling of protease-activated receptors
Source: Cell Res. 2024 Jul 12;34(10):725–34. doi: 10.1038/s41422-024-00997-2 (PMC11443083; doi:10.1038/s41422-024-00997-2)
Supplement: Supplementary file 13 — Supplementary information, Table S4 [file 41422_2024_997_MOESM13_ESM.pdf]

**Table S4. Thrombin–induced $G_q$ dissociation of WT and mutant PAR1.**

| | Efficacy±SEM[a,b] (%WT) | Expression (%WT) |
|---|---|---|
| WT | 100 | 100 |
| $F87^{N\text{-}term}A$ | $98.54 \pm 1.15^{NS}$ | 148.03±2.03 |
| $I88^{N\text{-}term}A$ | $49.75 \pm 2.91^{****}$ | 136.45±6.57 |
| $S89^{N\text{-}term}A$ | $76.98 \pm 4.57^{***}$ | 144.90±11.98 |
| $Y95^{N\text{-}term}A$ | $99.98 \pm 2.92^{NS}$ | 134.22±5.19 |
| $H255^{ECL2}A$ | $92.00 \pm 3.68^{NS}$ | 140.88±11.98 |
| $D256^{ECL2}A$ | $49.34 \pm 3.86^{****}$ | 124.06±14.09 |
| $V257^{ECL2}A$ | $74.66 \pm 4.12^{**}$ | 101.43±12.43 |
| $L258^{ECL2}A$ | $107.90 \pm 4.85^{NS}$ | 89.59±12.95 |
| $E260^{ECL2}A$ | $101.61 \pm 1.66^{NS}$ | 138.77±5.45 |
| $H336^{6.58}A$ | $95.02 \pm 6.85^{NS}$ | 148.75±7.28 |
| $Y337^{6.59}A$ | $58.72 \pm 0.34^{****}$ | 112.72±4.10 |
| $E347^{7.29}A$ | $59.65 \pm 2.05^{****}$ | 157.94±10.28 |
| $Y350^{7.32}A$ | $49.30 \pm 4.11^{****}$ | 87.80±8.22 |

[a]NanoBiT results of $G_q$ protein dissociation for PAR1 (WT and mutant) were normalized to the maximal response of wild-type PAR1. The data are presented as means ± SEM from at least three independent experiments performed in technical triplicate. $^{NS}P > 0.05$, $^{*}P < 0.05$, $^{**}P < 0.01$, $^{***}P < 0.001$ and $^{****}P < 0.0001$ by one-way ANOVA followed by Fisher's LSD multiple comparisons test compared with WT PAR1.

[b]The efficacy is defined as the window between the response induced by thrombin and the vehicle.
