# Supplementary material for: Structural basis of tethered agonism and G protein coupling of protease-activated receptors
Source: Cell Res. 2024 Jul 12;34(10):725–34. doi: 10.1038/s41422-024-00997-2 (PMC11443083; doi:10.1038/s41422-024-00997-2)
Supplement: Supplementary file 14 — Supplementary information, Table S5 [file 41422_2024_997_MOESM14_ESM.pdf]

Table S5. SFLLRN–induced G_q dissociation of WT and mutant PAR1.

	EC50 (μM) ±SEM[a]	pEC50±SEM[a]	ΔpEC50±SEM[a]	Efficacy±SEM[a,b] (%WT)	ΔEfficacy±SEM[a,b] (%WT)	Sample size	Expression (%WT)
WT	2.27±0.28	5.67±0.05	0	100	0	4	100
F182$^{3.32}$A	4.02±1.15^{NS}	5.44±0.15^{NS}	-0.19±0.03^{NS}	60.84±4.18****	-39.16±4.18****	3	100.10±13.03
Y183$^{3.33}$A	0.48±0.14^{NS}	6.36±0.14^{NS}	0.73±0.07**	60.15±4.42****	-39.85±4.42****	3	108.10±19.32
M186$^{3.36}$A	1.28±0.19^{NS}	5.90±0.07^{NS}	-0.18±0.06^{NS}	59.37±2.50****	-40.63±2.50****	3	104.30±5.78
F271$^{5.39}$A	82.47±14.72****	4.10±0.08****	-1.56±0.05****	43.16±0.77****	-55.84±0.77****	3	58.78±10.48
H336$^{6.58}$A	1.67±0.61^{NS}	5.87±0.22^{NS}	-0.34±0.12^{NS}	73.05±6.27**	-26.95±6.27**	4	88.83±17.20
Y337$^{6.59}$A	19.90±8.16^{NS}	4.88±0.27**	-1.19±0.23****	54.72±4.06****	-45.28±4.06****	3	54.56±11.01
Y353$^{7.35}$A	11.74±3.58^{NS}	4.97±0.13*	-0.69±0.01**	40.67±3.70****	-59.33±3.70****	3	70.35±16.75

[a]NanoBiT results of G_q protein dissociation for PAR1 (WT and mutant) were normalized to the maximal response of wild-type PAR1. The data are presented as means ± SEM from at least three independent experiments performed in technical triplicate. $^{NS}P > 0.05$, $^*P < 0.05$, $^{**}P < 0.01$, $^{***}P < 0.001$ and $^{****}P < 0.0001$ by one-way ANOVA followed by Fisher's LSD multiple comparisons test compared with WT PAR1.

[b]The efficacy is defined as the window between the maximal response (E_{max}) and the vehicle (no agonist).
