# Supplementary material for: Structural basis of tethered agonism and G protein coupling of protease-activated receptors
Source: Cell Res. 2024 Jul 12;34(10):725–34. doi: 10.1038/s41422-024-00997-2 (PMC11443083; doi:10.1038/s41422-024-00997-2)
Supplement: Supplementary file 15 — Supplementary information, Table S6 [file 41422_2024_997_MOESM15_ESM.pdf]

Table S6. SFLLRN–induced G_q dissociation of WT and mutant PAR1.

	EC50 (μM) ±SEM[a]	pEC50±SEM[a]	ΔpEC50±SEM[a]	Efficacy±SEM[a,b] (%WT)	ΔEfficacy±SEM[a,b] (%WT)	Sample size	Expression (%WT)
WT	1.58±0.10	5.81±0.03	0	100	0	4	100
K135$^{2.37}$A	1.18±0.17^{NS}	5.95±0.07^{NS}	0.09±0.07^{NS}	68.05±3.82***	-31.95±3.82***	4	45.27±7.27
P136$^{2.38}$A	1.27±0.47^{NS}	5.97±0.19^{NS}	0.18±0.13^{NS}	62.79±4.32****	-37.21±4.32****	3	51.83±5.83
M141$^{2.43}$A	1.46±0.37^{NS}	5.87±0.10^{NS}	0.17±0.14^{NS}	39.54±2.84****	-60.46±2.84****	4	50.14±14.09
D199$^{3.49}$A	7.13±1.67^{NS}	5.18±0.10**	-0.52±0.10^{NS}	46.86±4.08****	-53.14±4.08****	4	44.55±4.55
R200$^{3.50}$A	128.50±49.85****	4.10±0.31****	-1.89±0.16****	34.83±7.08****	-65.17±7.08****	4	51.77±4.64
V204$^{3.54}$A	1.68±0.25^{NS}	5.79±0.07^{NS}	0.03±0.06^{NS}	47.55±2.48****	-52.45±2.48****	3	114.00±15.60
P207$^{3.57}$A	1.53±0.40^{NS}	5.85±0.11^{NS}	0.09±0.12^{NS}	40.90±2.35****	-59.10±2.35****	3	52.87±7.77
M208^{ICL2}A	0.73±0.18^{NS}	6.16±0.11^{NS}	0.40±0.11^{NS}	41.37±3.18****	-58.63±3.18****	3	60.35±14.75
L211^{ICL2}A	2.26±0.38^{NS}	5.66±0.07^{NS}	0.13±0.08^{NS}	46.83±5.55****	-53.17±5.55****	3	103.70±11.34
S212^{ICL2}A	1.58±0.36^{NS}	5.83±0.10^{NS}	0.02±0.04^{NS}	80.26±6.17^{NS}	-19.74±6.17^{NS}	3	87.24±6.51
L297$^{5.65}$A	2.93±0.48^{NS}	5.51±0.07^{NS}	-0.32±0.06^{NS}	31.84±4.58****	-68.16±4.58****	4	71.37±14.13
V302^{ICL3}A	2.16±0.89^{NS}	5.76±0.16^{NS}	0.13±0.07^{NS}	71.94±6.29**	-28.06±6.29**	4	77.42±11.73
N304^{ICL3}A	2.00±0.49^{NS}	5.73±0.09^{NS}	0.05±0.04^{NS}	73.01±4.37**	-26.99±4.37**	3	67.45±1.27
R305^{ICL3}A	1.12±0.08^{NS}	5.96±0.03^{NS}	0.05±0.04^{NS}	83.63±2.07^{NS}	-16.37±2.07^{NS}	3	75.53±8.60
S306^{ICL3}A	5.08±3.24^{NS}	5.51±0.23^{NS}	-0.20±0.19^{NS}	67.65±0.53***	-32.25±0.53***	3	59.80±8.636
K308^{ICL3}A	1.32±0.08^{NS}	5.88±0.03^{NS}	-0.03±0.04^{NS}	54.79±2.15****	-45.21±2.15****	3	74.63±6.42
A311$^{6.33}$G	2.80±0.28^{NS}	5.56±0.04^{NS}	0.03±0.05^{NS}	61.82±3.48****	-38.18±3.48****	3	99.24±12.56
Y371$^{7.53}$A	46.45±19.00^{NS}	4.49±0.22****	-1.20±0.23****	29.39±7.51****	-70.61±7.51****	4	59.84±16.45
S375$^{8.47}$A	104.10±45.58****	4.16±0.25****	-1.51±0.26****	18.58±4.40****	-81.42±4.40****	4	19.66±1.02

[a]NanoBiT results of G_q protein dissociation for PAR1 (WT and mutant) were normalized to the maximal response of wild-type PAR1. The data are presented as means ± SEM from at least three independent experiments performed in technical triplicate. $^{NS}P > 0.05$, $^{*}P < 0.05$, $^{**}P < 0.01$, $^{***}P < 0.001$ and ****P <

0.0001 by one-way ANOVA followed by Fisher's LSD multiple comparisons test compared with WT PAR1.

[b]The eficacy is defined as the window between the maximal response (E_{max}) and the vehicle (no agonist).
