# Supplementary material for: Structural basis of tethered agonism and G protein coupling of protease-activated receptors
Source: Cell Res. 2024 Jul 12;34(10):725–34. doi: 10.1038/s41422-024-00997-2 (PMC11443083; doi:10.1038/s41422-024-00997-2)
Supplement: Supplementary file 16 — Supplementary information, Table S7 [file 41422_2024_997_MOESM16_ESM.pdf]

Table S7. SFLLRN–induced G_i dissociation of WT and mutant PAR1.

	EC50 (μM) ±SEM[a]	pEC50±SEM[a]	ΔpEC50±SEM[a]	Efficacy±SEM[a,b] (%WT)	ΔEfficacy±SEM[a,b] (%WT)	Sample size	Expression (%WT)
WT	0.36±0.05	6.48±0.06	0	100	0	6	100
K135$^{2.37}$A	2.07±0.27^{NS}	5.69±0.06****	-0.72±0.07****	82.28±0.43**	-17.72±0.43**	3	61.03±6.32
P136$^{2.38}$A	0.57±0.07^{NS}	6.25±0.05^{NS}	-0.30±0.01^{NS}	97.40±0.43^{NS}	-2.60±0.43^{NS}	3	80.04±4.66
M141$^{2.43}$A	0.62±0.08^{NS}	6.22±0.05^{NS}	-0.44±0.03***	95.56±3.85^{NS}	-4.44±3.85^{NS}	5	79.02±13.90
D199$^{3.49}$A	3.49±0.63^{NS}	5.48±0.07****	-1.22±0.06****	83.70±5.00**	-16.30±5.00**	4	90.63±12.36
R200$^{3.50}$A	44.48±10.20****	4.38±0.09****	-1.80±0.10****	22.56±3.21****	-77.44±3.21****	4	94.14±18.00
V204$^{3.54}$A	1.75±0.18^{NS}	5.76±0.05****	-0.79±0.04****	76.43±3.21***	-23.57±3.21***	3	96.19±12.57
P207$^{3.57}$A	1.27±0.06^{NS}	5.90±0.02***	-0.63±0.06****	60.21±3.16****	-39.79±3.16****	3	57.61±7.71
M208^{ICL2}A	0.27±0.03^{NS}	6.58±0.04^{NS}	0.07±0.03^{NS}	95.95±1.35^{NS}	-4.05±1.35^{NS}	3	76.5±7.62
L211^{ICL2}A	1.04±0.03^{NS}	6.02±0.13*	-0.22±0.08^{NS}	90.98±4.51^{NS}	-9.02±4.51^{NS}	3	153.4±15.32
S212^{ICL2}A	0.69±0.11^{NS}	6.17±0.07^{NS}	-0.10±0.02^{NS}	96.96±3.96^{NS}	-3.04±3.96^{NS}	3	101.10±14.44
L297$^{5.65}$A	1.35±0.14^{NS}	5.87±0.04***	-0.63±0.07****	30.66±2.69****	-69.34±2.69****	3	77.56±4.62
V302^{ICL3}A	0.37±0.06^{NS}	6.44±0.07^{NS}	-0.10±0.02^{NS}	96.67±0.86^{NS}	-3.33±0.86^{NS}	3	78.61±4.44
N304^{ICL3}A	0.41±0.08^{NS}	6.41±0.08^{NS}	-0.21±0.02^{NS}	99.16±2.17^{NS}	-0.84±2.17^{NS}	5	107.4±19.51
R305^{ICL3}A	0.36±0.06^{NS}	6.46±0.07^{NS}	-0.02±0.07^{NS}	101.90±3.05^{NS}	1.93±3.05^{NS}	3	80.67±9.03
S306^{ICL3}A	0.54±0.12^{NS}	6.31±0.09^{NS}	-0.33±0.03**	99.24±1.36^{NS}	-0.76±1.36^{NS}	5	113.60±11.95
K308^{ICL3}A	0.93±0.15^{NS}	6.04±0.08*	-0.44±0.05**	83.28±3.86*	-16.72±3.86*	3	105.20±8.10
A311$^{6.33}$G	1.17±0.26^{NS}	5.96±0.10**	-0.32±0.06*	87.71±2.37^{NS}	-12.29±2.37^{NS}	3	113.50±12.27
Y371$^{7.53}$A	14.19±2.52*	4.89±0.09****	-1.60±0.07****	47.79±3.29****	-52.21±3.29****	6	96.09±15.08
S375$^{8.47}$A	22.09±7.59****	4.82±0.18****	-1.70±0.14****	30.46±2.23****	-69.54±2.23****	5	85.15±15.03

[a]NanoBiT results of G_i protein dissociation for PAR1 (WT and mutant) were normalized to the maximal response of wild-type PAR1. The data are presented as means ± SEM from at least three independent experiments performed in technical triplicate. $^{NS}P > 0.05$, $*P < 0.05$, $**P < 0.01$, $***P < 0.001$ and $****P < 0.0001$

by one-way ANOVA followed by Fisher's LSD multiple comparisons test compared with WT PAR1.

[b]The efficacy is defined as the window between the maximal response (E_{max}) and the vehicle (no agonist).
